# Targeted Drug Delivery and Theranostic Strategies in Malignant Lymphomas

**DOI:** 10.3390/cancers14030626

**Published:** 2022-01-26

**Authors:** Tomas Etrych, Alena Braunova, David Zogala, Lukas Lambert, Nicol Renesova, Pavel Klener

**Affiliations:** 1 Institute of Macromolecular Chemistry, Czech Academy of Sciences, 162 06 Prague, Czech Republic; etrych@imc.cas.cz (T.E.); braunova@imc.cas.cz (A.B.); 2 Institute of Nuclear Medicine, General University Hospital and First Faculty of Medicine, Charles University in Prague, 128 08 Prague, Czech Republic; david.zogala@vfn.cz; 3 Department of Radiology, General University Hospital and First Faculty of Medicine, Charles University in Prague, 128 08 Prague, Czech Republic; lukas.lambert@vfn.cz; 4 First Faculty of Medicine, Institute of Pathological Physiology, Charles University, 121 08 Prague, Czech Republic; nicol.renesova@lf1.cuni.cz; 5 First Department of Internal Medicine-Hematology, General University Hospital and First Faculty of Medicine, Charles University in Prague, 128 08 Prague, Czech Republic

**Keywords:** targeted drug delivery, theranostics, lymphoma, antibody–drug conjugates, liposomes, nanomedicine, nuclear imaging, magnetic resonance imaging

## Abstract

**Simple Summary:**

The concept of targeted drug delivery (TDD) represents an innovative and effective treatment approach, which was developed with an attempt to minimize damage toward healthy tissues. Radioimmunotherapy (RIT) with radioimmunoconjugates and TDD with antibody–drug conjugates (ADC) both represent drug delivery systems (DDS) based on monoclonal antibody-mediated delivery of toxic payloads toward the lymphoma tissue. Other modalities of TDD are based on new formulations of “old” cytostatic agents and their passive trapping in the tumor bulk by means of enhanced permeability and retention (EPH) effect. These comprise several clinically approved liposomal formulations of anthracyclines and many investigational nanomedicines including pegylated and non-pegylated liposomes, or polymer-based nanoparticles. Currently, the diagnostic and restaging procedures in aggressive lymphomas are based on nuclear imaging, predominantly on 2-[F^18^] fluoro-2-deoxy-D-glucose (FDG) positron emission tomography (PET). On a preclinical level, it has been repeatedly demonstrated that the assessment of response and therapy delivery can be fused. Such a theranostic approach that would combine the diagnostic or restaging imaging procedure with a targeted therapy represents an appealing innovative strategy in personalized medicine in hemato-oncology.

**Abstract:**

Malignant lymphomas represent the most common type of hematologic malignancies. The first clinically approved TDD modalities in lymphoma patients were anti-CD20 radioimmunoconjugates (RIT) ^131^I-tositumomab and ^90^Y-ibritumomab-tiuxetan. The later clinical success of the first approved antibody–drug conjugate (ADC) for the treatment of lymphomas, anti-CD30 brentuximab vedotin, paved the path for the preclinical development and clinical testing of several other ADCs, including polatuzumab vedotin and loncastuximab tesirine. Other modalities of TDD are based on new formulations of “old” cytostatic agents and their passive trapping in the lymphoma tissue by means of the enhanced permeability and retention (EPR) effect. Currently, the diagnostic and restaging procedures in aggressive lymphomas are based on nuclear imaging, namely PET. A theranostic approach that combines diagnostic or restaging lymphoma imaging with targeted treatment represents an appealing innovative strategy in personalized medicine. The future of theranostics will require not only the capability to provide suitable disease-specific molecular probes but also expertise on big data processing and evaluation. Here, we review the concept of targeted drug delivery in malignant lymphomas from RIT and ADC to a wide array of passively and actively targeted nano-sized investigational agents. We also discuss the future of molecular imaging with special focus on monoclonal antibody-based and monoclonal antibody-derived theranostic strategies.

## 1. Introduction

### 1.1. Malignant Lymphomas

Malignant lymphomas are heterogeneous clonal lymphoid tumors that develop from a malignant transformation of precursor or peripheral lymphocytes during various states of their differentiation [1]. Malignant lymphomas comprise Hodgkin lymphomas (HL), rare aggressive lymphomas of B-cell origin, and non-Hodgkin lymphomas (NHL). NHL represent the most common type of hematologic malignancies with an incidence of approx. 13 per 100 thousand inhabitants in the Western Hemisphere. Aggressive B-NHL comprise diffuse large B-cell lymphoma (DLBCL), the most common type of NHL, and many other lymphoma subtypes, including mantle cell lymphoma (MCL), or Burkitt lymphoma. Indolent lymphomas include follicular lymphoma (FL), the second most prevalent lymphoma subtype, marginal zone lymphoma (MZL), and many other lymphoma subtypes. T-NHL consist of a heterogeneous group of malignancies with usually aggressive biological behavior. T-NHL can be roughly divided into systemic (nodal), leukemic, and cutaneous subtypes [2,3].

Front-line therapy of malignant lymphomas is still based on chemotherapy, i.e., on standard genotoxic cytostatic drugs, e.g., alkylating agents (cyclophosphamide, cisplatin), anthracyclines (doxorubicin), nucleoside analogues (cytarabine, fludarabine), vinca alkaloids (vincristine), topoisomerase inhibitors (etoposide), and other DNA-damaging agents (bleomycin). In the case of CD20-positive B-NHL, the chemotherapy is combined with anti-CD20 monoclonal antibodies, rituximab or obinutuzumab, while CD30-positive T-cell lymphomas are treated with the combination of conventional chemotherapy and anti-CD30 antibody–drug conjugate brentuximab vedotin [4,5].

Salvage therapy of aggressive NHL also largely relies on standard salvage polychemotherapy regimens, in indicated cases followed by the consolidation with high-dose therapy and autologous stem cell transplantation [6]. In recent years, several new treatment options emerged for the patients with advanced relapses of aggressive NHL. New anti-CD19 antibody tafasitamab and new antibody–drug conjugates, anti-CD79B polatuzumab vedotin and anti-CD19 loncastuximab tesirine, were approved for patients with relapsed or refractory (R/R) DLBCL [7,8]. Bruton tyrosine-kinase inhibitor ibrutinib has significantly prolonged survival parameters of patients with R/R MCL [9,10]. Immunomodulatory agent lenalidomide has been approved for patients with FL or MCL [11]. Anti-CD30 antibody–drug conjugate brentuximab vedotin and immune check point inhibitors (nivolumab, pembrolizumab) have been approved for salvage therapy of R/R HL [12,13,14]. Most importantly, patients with R/R DLBCL or MCL can be offered adoptive immunotherapy based on ex vivo expanded autologous T-cells with genetically engineered chimeric antigen receptors (CARs) targeted against CD19 antigens (CAR19 T-cells) [15,16,17,18]. CAR19 T-cell-based therapy can induce long-term remissions in heavily pre-treated patients with adverse morphology or molecular-cytogenetic aberrations.

### 1.2. The Concept of Targeted Drug Delivery and Controlled Drug Release

**Figure 1:**
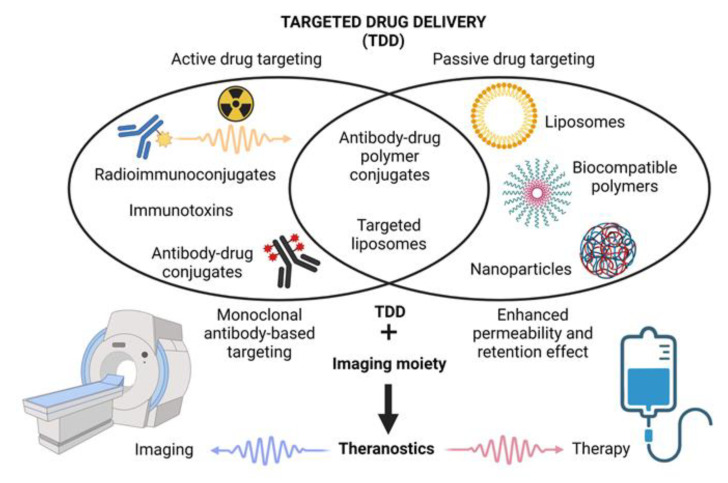
Targeted drug delivery systems and theranostics (Created with BioRender 20 December 2021).

In 1906, a German pathologist, immunologist, and Nobel Prize laureate Paul Ehrlich formulated the requirements for a new type of an ideal drug called “a magic bullet”, which would deliver the anti-cancer drug directly to the tumor with minimal release of the free drug in the bloodstream during transport [19,20]. Currently, three established large groups of anti-cancer agents fulfill the original idea of the magic bullet proposed by Paul Ehrlich: (1.) actively targeted biologicals based on monoclonal antibody-mediated delivery of toxic warheads (ADC, RIT), (2.) passively targeted DDS based on the enhanced permeability and retention (EPR) effect (liposomes, polymeric nanocarriers, and other types of nanomedicines), and (3.) overlapping agents (various types of actively targeted nanomedicines). The addition of an imaging moiety besides the toxic warheads can convert these agents into theranostics (Figure 1).

## 2. Antibody–Drug Conjugates (ADCs) and Immunotoxins: MAb-Mediated TDD

**Figure 2:**
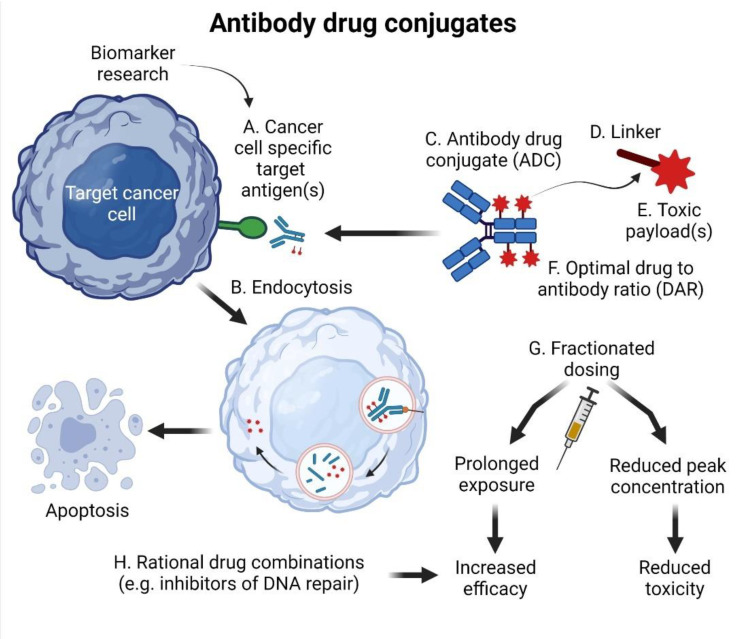
Mode of action of currently approved ADCs and the potential improvement of next-generation ADCs. The efficacy of next-generation ADCs might be impacted by the following features: (**A**) identification of suitable cancer cell-specific antigens; (**B**) development of ADCs that will foster receptor-mediated endocytosis; (**C**) modification of MAbs that would improve the pharmacokinetics of ADC; (**D**) innovative linkers that would induce cancer cell-specific cleavage of (**E**) more effective payloads bound with (**F**) increased DAR; (**G**) optimized dosing schedules; (**H**) rational drug combinations. Created with BioRender 20 December 2021.

Monoclonal antibodies (MAbs) were the first targeted biologicals approved for the clinical practice in hemato-oncology [21,22]. The unprecedented success of anti-CD20 rituximab paved the way for the development of next-generation and glycoengineered monoclonal antibodies [8,23]. Naked MAbs exert their anti-tumor activity predominantly via activation of host immunity, namely complement (complement-dependent cytotoxicity, CDC) and immune cells, including macrophages and natural killer (NK) cells (antibody-dependent cell-mediated cytotoxicity, ADCC, antibody-dependent cell-mediated phagocytosis, ADCP) [24]. Besides that, however, MAbs represent ideal biological moieties for TDD including RIT and ADC [25]. The concept of ADC is depicted in Figure 2.

### 2.1. The Structure of ADCs

For the best clinical activity and minimal toxicity, the ADC must fulfill several criteria [26]. First, the target antigen should be highly (ideally exclusively) expressed on tumor cells, and the binding of ADC to the antigen should trigger receptor-mediated endocytosis of the ADC–antigen complex(es) (Figure 2A,B). The low rate of receptor-mediated endocytosis is one of the reasons why the CD20 antigen is not a suitable target for ADC-based strategy (but remains a suitable target for RIT).

Second, to minimize adverse side effects, the linker (Figure 2C,D) between the antibody and the toxic payload should be leak-proof [20]. Specifically, it should ensure a controlled drug release at the tumor site but not during transport in the bloodstream. This is achieved, for example, by enzymatic cleavage by cathepsin B, which recognizes dipeptide sequences such as valine–citrulline or by pH-sensitive hydrolytic cleavage of the hydrazone (acid-labile) linkers [27].

The toxic payloads (Figure 2E) comprise various small molecules with high cytostatic potency, usually antimitotic and alkylating agents. The mitotic poisons include maytansine derivatives derived from the plant *Maytenus ovatus* DM1 (emtansine, mertansine) and DM4 (soravtansine, ravtansine) and auristatins derived from the marine gastropod *Dolabella auricularia* (monomethylauristatin E/vedotin/MMAE, monomethylauristatin F/mafodotin/MMAF)). Alkylating agents include calicheamycin, pyrrolobenzodiazepine dimer (PBD), doxorubicin, and other agents [28].

Third, the ADC should be loaded with optimal numbers of toxic payloads, which is expressed as a drug-to-antibody ratio (DAR) (Figure 2F). DAR impacts key physico-chemical, pharmacokinetic, and pharmacodynamic features of the ADC. The currently approved ADCs have a DAR between three and four.

Due to the intentional modulation of the MAb molecule, the immune functions of the antibody carriers within ADCs usually have suppressed immune functions (compared to the parental free antibodies). The other reason why ADCs act as poor immunotherapeuticals is the dosing, which is on average 5–10 times lower compared to that of naked MAbs (e.g., anti-CD20 rituximab 375–500 mg/m^2^, anti-CD20 obinutuzumab 1000 mg flat dose, anti-CD79b polatuzumab vedotin 1.8 mg/kg). As a result of their modified structure and lower dosing, the mode of action of ADCs is almost exclusively based on TDD, while their roles as mediators of CDC, ADCC, and ADCP are to a great extent suppressed.

### 2.2. Gemtuzumab Ozogamicin (GO): The First Global Approval

The first ADC approved in clinical practice was gemtuzumab ozogamicin (GO), an anti-CD33 antibody conjugated with calicheamicin [29]. GO was granted accelerated approval in 2000 for the therapy of R/R acute myeloid leukemia (AML) at the dose of 9 mg/m^2^ at days one and 15, but in 2010 the marketing approval was voluntarily withdrawn because of reported high systemic toxicity. In 2017, GO was regranted approval with modified dosing at a lower dose “fractionated” schedule of 3 mg/m^2^ days at one, four, and seven [30]. GO is currently being evaluated in numerous clinical trials in combination with chemotherapy or targeted agents for patients with newly diagnosed and treatment-refractory AML, including CPX-351 (NCT03904251), venetoclax (NCT04070768), arsenic trioxide plus all trans-retinoic acid (NCT01409161), and many others.

### 2.3. Brentuximab Vedotin (BV): The First Clinical Approval for the Therapy of Lymphomas

The second clinically approved ADC was anti-CD30 brentuximab vedotin for the therapy of lymphomas (Table 1).

BV is conjugated with the synthetic antitubulin molecule monomethyl auristatin E (MMAE). In 2011, BV was approved for the therapy of relapsed/refractory (R/R) Hodgkin lymphomas and R/R anaplastic large T-cell lymphomas (ALCL), both characterized by aberrant (over)expression of CD30 [31]. In 2017, BV was approved for the therapy of R/R cutaneous T-cell lymphomas based on the results of the ALCANZA phase III clinical trial, which demonstrated significant improvement in objective response rates achieved in patients treated with BV versus physician’s choice of methotrexate or bexarotene [32]. In 2018, BV in combination with chemotherapy was approved for the therapy of patients with so far untreated Hodgkin lymphoma based on the results of the ECHELON-1 study [33]. Based on the ECHELON-2 phase III clinical trial, BV in combination with cyclophosphamide, doxorubicin, and prednisone was approved for first-line therapy of CD30+ peripheral T-cell lymphomas [5]. The success of BV clearly demonstrated that the concept of ADC can be effective even in the case when the naked antibody (anti-CD30 SGN-30) exerted virtually no clinical activity [34]. The loss of immune-mediated activity of SGN-35 (i.e., SGN-30 conjugated with MMAE) did not hamper the anti-tumor activity of BV, because the MoA of BV is fundamentally different compared to the naked SGN-30 MAb. It must be underlined that CD30 represents an ideal target antigen for the ADC-mediated delivery of a mitotic poison for two reasons. First, CD30 is aberrantly expressed almost exclusively on lymphoma cells but not on healthy tissues. Second, CD30 is readily internalized upon binding of BV, thus delivering the toxic payload specifically within lymphoma cells. Currently, BV is being evaluated in clinical trials for patients with T-cell lymphomas in combination with chemotherapy (NCT05006664), pembrolizumab (NCT04795869), lenalidomide (NCT03302728), and other drug combinations.

### 2.4. Inotuzumab Ozogamicin: Targeting B-Cell Acute Lymphoblastic Leukemia/Lymphoma

CD22 is a glycoprotein expressed on B-cell acute lymphoblastic leukemia (B-ALL) blasts. Inotuzumab ozogamicin (InO) is a humanized anti-CD22 monoclonal antibody conjugated to calicheamicin [35]. In June 2017, InO was granted approval for the treatment of patients with R/R CD22-positive B-ALL who have failed at least one tyrosine kinase inhibitor-based therapy [36,37]. Of note, InO is effective also in challenging populations of patients with high baseline disease burden including extramedullary disease or lymphoblastic lymphoma [38]. However, a phase III randomized trial in patients with R/R CD20/CD22-positive aggressive B-NHL failed to demonstrate a benefit of InO plus rituximab compared to the investigator’s choice (R-bendamustine or R-gemcitabine) [39].

### 2.5. Polatuzumab Vedotin (PV): The Game Changer in DLBCL?

CD79B is expressed on B-cells as a part of the B-cell receptor. Polatuzumab vedotin (PV), in combination with bendamustine and rituximab (PV-BR), was approved in 2019 for the therapy of R/R DLBCL based on the results of a phase II clinical trial (NCT02257567) [40]. The ORR in the PV-BR and BR arms was 63% and 25%, respectively. The currently running POLARIX phase III trial is comparing the efficacy of PV in combination with rituximab and chemotherapy (cyclophosphamide, doxorubicin, and prednisone (CHP)) versus rituximab and CHOP (CHP plus vincristine) in previously untreated patients with DLBCL patients. A total of 879 patients were randomized 1:1 to receive PV-R-CHP (+vincristine placebo) or R-CHOP (+PV placebo). The PV-R-CHP combination demonstrated a 27% reduction in the relative risk of disease progression, relapse, or death compared with R-CHOP, with a similar safety profile [41]. PV-R-CHP is thus the first regimen in the last 20 years to significantly improve outcomes in previously untreated DLBCL. PV is currently being evaluated in combination with salvage chemotherapy for patients with R/R DLBCL (NCT04182204, NCT04665765). PV-BR, and PV in combination with lenalidomide and rituximab, is being evaluated in two clinical trials in patients with R/R MCL (NCT04913103, NCT04659044). PV-R-CHP in combination with a bispecific antibody glofitamab is being studied in patients with newly diagnosed high-risk DLBCL (NCT04914741), while a dose-reduced PV-R-CHP regimen is currently being tested in elderly patients with so far untreated DLBCL (NCT04594798).

### 2.6. Loncastuximab Tesirine (LT): Another Player in CD19-Directed Strategies

CD19-directed therapies became a mainstream of T-cell engaging immunotherapies including adoptive cellular therapy with CD19-directed T-cells with chimeric antigen receptors (axicabtagene ciloleucel, tisagenlecleucel, lisocabtagene maraleucel, brexucabtagene autoleucel), bispecific T-cell engagers (blinatumomab), or CD19-directed naked monoclonal antibodies (tafasitamab) [8,15,16,17,18,42]. In addition, CD19 represents an appealing target antigen for TDD [43]. First, although CD19 is expressed on all mature B-cells, it is not targeted by currently used front-line immunotherapy approaches based on anti-CD20 antibodies. Second, contrary to CD20, CD19 antigens are internalized upon the binding of anti-CD19 antibodies. CD19 antibody conjugated to tesirine, a pyrrolobenzodiazepine dimer (PBDD) cytotoxin, loncastuximab-tesirine (LT), demonstrated promising clinical activity in prognostically adverse R/R DLBCL including double-hit and transformed lymphoma in the LOTIS-2 clinical trial [44]. The overall response rate was 48.3% with 24.1% complete remissions (CRs). PBDD-associated adverse events including oedema, effusions, rash, and liver enzyme elevations were successfully managed by improved supportive care and dose reduction. LT is currently being tested in several clinical trials in patients with R/R NHL (in combination with venetoclax, NCT05053659), with R/R follicular lymphoma (in combination with rituximab, NCT04998669), in R/R MCL and DLBCL (in combination with ibrutinib, NCT03684694), in R/R B-NHL (in combination with salvage chemotherapy, LOTIS-7, NCT04970901), in newly diagnosed DLBCL (in combination with R-CHP, LOTIS-8, NCT03684694), or in newly diagnosed frail/unfit (elderly) DLBCL patients (in combination with rituximab, LOTIS-9, NCT05144009).

### 2.7. Next-Generation ADCs

The ADCs were developed as sophisticated targeted drug delivery biologicals that improved the therapeutic index of various toxic substances [45]. Besides targeted drug delivery, further improvement of the therapeutic index was achieved by tumor-mediated linker cleavage, e.g., by cathepsin B, which recognizes dipeptide sequences such as valine–citrulline [46]. From this perspective, ADCs can be regarded as prodrugs, actively delivered into the tumor, and fully activated by the tumor. Further development (or evolution) of ADC biotechnology will focus on all parts of the ADC macromolecule, including the MAb carrier, the linker, and the toxic payload (Figure 2). Besides the ADC itself, two other critical factors will impact the efficacy and safety of next-generation ADCs: (1.) selection of suitable tumor-specific or tumor-associated antigen(s), and (2.) optimal dosing schedules including fractionated dosing of the currently approved ADCs [47].

Receptor tyrosine kinase-like orphan receptor 1 (ROR1) represents a promising target antigen, because it is expressed on leukemia and lymphoma cells but not on healthy tissues [48]. Several anti-ROR1 antibodies and ADCs are currently in various stages of preclinical development. VLS-101, anti-ROR1 humanized IgG1 MAb conjugated with MMAE via a standard maleimidocaproyl-valine-citrulline-para-aminobenzoate linker, demonstrated anti-tumor efficacy on patient-derived xenograft models of Richter syndrome (transformation of chronic lymphocytic leukemia to aggressive lymphoma) [49].

Decreased systemic toxicity may be achieved by genetic modification of the Fc fragment of the MAb carrier with an attempt to attenuate the non-specific binding of the Fc fragment to Fcγ receptors expressed on the cells of the reticuloendothelial system, which is responsible for some of the side effects including liver toxicity and myelosuppression. Conditional activation of the binding moiety of the ADC in the tumor tissue by active antibody masking can further increase efficacy and decrease the toxicity of the innovative ADC platforms (e.g., SAFEbody) [50].

Innovative linker technologies will allow bioengineering of ADC constructs with significantly increased DAR (from the usual 3–4 to >10–20). For example, the fleximer linker used in upifitamab rilsodotin (XMT-1536), an ADC directed against sodium-dependent phosphate transport protein 2 (BNaPi2b) and loaded with auristatin, is a biodegradable, highly biocompatible, water-soluble polymer, to which are attached multiple molecules of the auristatin drug yielding a DAR between 12 and 15 [51]. The toxic warheads will broaden beyond antimicrotubule agents to other potent cytotoxic or targeted drugs including topoisomerase inhibitors (e.g., exatecan), alkylating agents (e.g., pyrrolobenzodiazepines), BH3 mimetics (e.g., BCL2 inhibitors), or immunostimulants (e.g., toll-like receptor agonists) [52]. For example, huXBR1-402-G5-PNU, a novel anti-ROR1 ADC, is loaded with a highly potent anthracycline derivative of nemorubicin (PNU-159682) and demonstrated promising clinical activity in preclinical models of MCL after the failure of CAR19 T-cell-based therapy [53].

### 2.8. Immunotoxins: From Denileukin Diftitox to Moxetumomab Pasudotox and Beyond

Recombinant immunotoxins (IT) are genetically engineered proteins composed of a targeting moiety fused to a bacterial toxin, usually an enzymatically active portion of the diphtheria toxin fragments. The most frequently used targeting moieties of recombinant ITs comprise the fragment variable (Fv), the antibody-binding (Fab) portion of a monoclonal antibody, or the receptor-binding domain of human interleukin 2 (IL-2).

Denileukin diftitox is a diphtheria fusion protein composed of the truncated diphtheria toxin and human IL-2. Denileukin diftitox was approved by the FDA in 1999 for the treatment of cutaneous T-NHL, but it is not available on the market [54]. Next-generation IL-2 cytotoxic fusion protein E7777 has shown clinical activity in Japanese patients with R/R T-NHL [55].

Moxetumomab pasudotox is composed of the Fv fragment of an anti-CD22 monoclonal antibody fused to a 38 kDa fragment of Pseudomonas exotoxin A, PE38 [56]. In 2018, the FDA approved moxetumomab pasudotox-tdfk for patients with hairy cell leukemia who failed two lines of systemic therapy [57].

## 3. Liposomes, Polymeric Nanocarriers and Other Types of Nanomedicines

### 3.1. Nanosystems for TDD: Structure, Passive and Active Targeting

Thanks to huge progress in the field of biomedical polymer research, Ehrlich’s original idea of the magic bullet gave rise to a new scientific discipline called controlled drug delivery, or controlled drug release. Various types of targeted DDS, such as liposomes, polymeric nanocarriers, polymeric micelles, solid lipid nanocarriers, protein-based nanocarriers, dendrimers, carbon nanotubes, or magnetic nanoparticles, are currently being studied with the aim to improve and enhance the effectiveness of the treatment of serious diseases such as cancer, inflammatory diseases, or bacterial infections. The anti-cancer activity, as well as toxic side effects of these therapeutics, is strongly dependent on their molecular weight, structure, hydrodynamic size, charge, surface properties and functionality, type of bond used for the drug attachment (enzymatically or hydrolytically cleavable), rate of cellular uptake, and the biological behavior, such as biocompatibility, uptake by the immune system (macrophages), and circulation time in the bloodstream.

One of the unique properties of all TDD nanosystems is their passive tumor targeting mediated by the enhanced permeability and retention (EPR) effect, first described by Matsumura and Maeda in 1986. The EPR effect is based on the accumulation of macromolecules within solid tumors due to anatomical differences between the structure of the healthy (organized) and tumor (disorganized) vasculature and lymphatic drainage [58]. Active targeting of nanosystems can be achieved by the attachment of specific ligands (e.g., antibodies, peptides, vitamins, or hormones) with the ability to bind to cognate receptors highly (or exclusively) expressed on the surface of target cells (Figure 1). Nanosystems can also serve as prodrugs that are activated by unique features of the tumor microenvironment including pH, enzymatic activity, temperature, or osmolality.

### 3.2. Liposomal Formulations of Cytostatics

**Figure 3:**
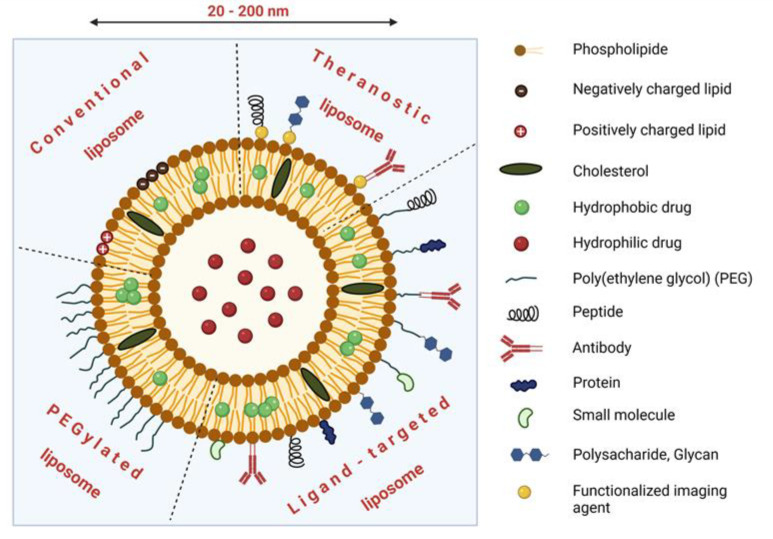
The structure of liposome-based nanocarriers. Created with BioRender 20 December 2021.

Liposomes were one of the earliest nanocarriers used for passive targeting of drugs and belong to the most clinically established nanocarriers for therapeutic agent delivery up to now. Liposomes are artificial spherical bilayer vesicles prepared from insoluble polar lipids, e.g., naturally derived phospholipids [59,60]. Under aqueous conditions, they assemble into highly organized membrane-forming aggregates with a structure such as biological membranes, where an aqueous core domain is surrounded by the lipid bilayer [61,62]. Due to the extreme versatility of liposomes and their amphiphilic character, the loaded drugs can be entrapped within the liposome not only in the hydrophilic cavity but also in the lipid bilayer (see Figure 3) [63,64,65,66].

Uptake of the drug inside the liposome contributes significantly to an increased therapeutic index of the loaded drugs, mainly due to (i) protection of the drug against enzymatic degradation, immunologic and chemical inactivation; (ii) prevention of its metabolization before reaching target tissue; (iii) reduced drug exposure to healthy tissues; (iv) increased blood circulation half-life [67,68]. According to the number of their layers (also called lamellae), the liposomes can be divided into multilamellar (>500 nm), small unilamellar (20–100 nm), or large unilamellar vesicles (>100 nm) [59]. In general, the lipids utilized for liposome preparation are natural and synthetic double-chain lipids (phosphorus polar head and glycerol backbone) and sterols, especially cholesterol [69].

Non-coated liposomes are often cleared by the cells of the reticuloendothelial system, which is the reason why there was an effort to modify their surface with an inert biocompatible hydrophilic polymer. A polyethylene glycol (PEG) is predominantly employed as a non-toxic, non-ionic, biocompatible molecule for PEG modification, so-called PEGylation [66]. PEGylation protects liposomes from immune-mediated clearance and confers higher stability, which leads to prolonged circulation of the PEGylated liposomes in the organism [70]. A coating of the liposomes with glycoproteins, oligosaccharides, and polysaccharides was also studied with the aim to prevent the liposomal blood clearance [71]. The surface-modified liposomes are called stealth liposomes and can be prepared with PEG chains of various length covalently attached to the hydrocarbon chain anchors.

### 3.3. Liposomal Formulations of Anthracyclines

#### 3.3.1. Liposomal Formulations of Doxorubicin—The First Nanomedicines Approved for the Treatment of Cancer

Anthracyclines including doxorubicin, daunorubicin, or epirubicin belong to backbone cytostatic agents for the treatment of both solid tumors and hematological malignancies [65,72]. Liposomes have been primarily studied as anthracycline carriers to reduce the anthracycline-associated cardiotoxicity and increase their therapeutic index [73]. Two liposomal formulations of doxorubicin have been granted marketing approval up to the present: non-PEGylated liposomal doxorubicin (NPLD; Dox citrate-encapsulated formulation; Myocet^®^), and PEGylated liposomal doxorubicin (PLD; Caelyx^®^ /Doxyl^®^/LipoDox^®^) [66,68,69,74]. In 1995, PLD was the first liposomal drug class of agent approved for the treatment of AIDS-related Kaposi’s sarcoma [75]. Besides Kaposi’s sarcoma, PLD was approved for the treatment of patients with ovarian cancer (in 1998) and metastatic breast cancer (in 2003). In 2007, PLD (in combination with bortezomib) was approved for patients with MM who have received at least one prior therapy not including bortezomib [74].

The second approved liposomal formulation of doxorubicin, NPLD (Myocet), was approved in the year 2000 in combination with cyclophosphamide for first-line therapy of metastatic breast cancer.

The non-PEGylated liposomal formulation of daunorubicin, DaunoXome^®^ (DNX), is indicated as a first-line cytotoxic therapy for advanced HIV-associated Kaposi’s sarcoma.

CPX-351 (Vyxeos^®^) is a liposome-based nanocarrier encapsulating daunorubicin and cytarabine in 1:5 molar ratio [76]. Therapy with CPX-351 was associated with significantly prolonged survival compared to the standard of care “7 + 3” regimen (conventional cytarabine and daunorubicin) in the elderly patients with so far untreated AML, while its safety profile was comparable [77,78]. In 2021, Vyxeos liposomal^®^ was approved also for the treatment of secondary AML in pediatric patients [79].

#### 3.3.2. Liposomal Formulations of Doxorubicin in the Therapy of Aggressive Lymphomas

The rationale for the use of liposomal formulations of doxorubicin in patients with aggressive lymphoma is based on two presumptions. First, doxorubicin is a key component of the R-CHOP-like regimens, which still represent the current golden standard of care for first-line therapy of patients with most types of aggressive lymphomas but has been associated with cardiotoxicity, which limits its use in the cohort of frail/unfit patients. Second, replacement of free doxorubicin with the liposomal nanodrug would decrease cardiotoxicity of free doxorubicin, thereby offering access to the standard of care to more patients including the elderly and comorbid ones. Importantly, in a large meta-analysis of patients with DLBCL treated with NPLD, Visco et al. endorsed the value of NPLD both in terms of response and survival [80]. In contrast, although Sancho et al. confirmed the non-inferiority of NPLD to conventional doxorubicin as part of frontline immunochemotherapy in patients with so far untreated aggressive lymphomas, the substitution with NPLD was not associated with less early cardiotoxicity, although some reduced cardiac safety signals were observed [81]. Similarly, NPLD did not reduce cardiotoxicity, although cardiac safety signals were elevated in R-CHOP compared to R-COMP in an Austrian randomized phase III study [82]. So, the cardiotoxicity issue of the liposomal formulations of doxorubicin remains a matter of debate. Liposomal formulations of doxorubicin as part of various polychemo/immunotherapy regimens demonstrated promising activity with an acceptable safety profile in patients with classical Hodgkin lymphoma, primary mediastinal B-cell lymphoma, and other types of aggressive lymphomas [83,84]. NPLD in combination with romidepsin, a histone deacetylase inhibitor, demonstrated an acceptable safety profile and promising clinical efficacy with deep skin responses in relapsed/refractory cutaneous T-cell lymphomas [85]. Excellent anti-tumor efficacy was reported in a small cohort of R/R DLBCL patients treated with the combination of two liposomal nanodrugs: NPLD and albumin-bound paclitaxel (nabpaclitaxel, Abraxane) [86].

### 3.4. Liposomal Formulations of Vinca Alkaloids and Cytarabine

Like anthracyclines, vinca alkaloids belong to classical cytostatics broadly used in diverse hematologic malignancies. In 2012, the liposomal formulation of vincristine (vincristine sulphate liposome injection (VSLI), Marquibo^®^) was approved for therapy of Philadelphia chromosome negative (Ph-) acute lymphoblastic leukemias. This liposomal formulation reduced the side effects of free vincristine, especially neuropathy. VSLI was tested also in patients with aggressive lymphomas, where it was substituted for the conventional vincristine in the R-CHOP regimen (becoming R-CHMP). According to the published data, the R-CHMP combination was highly active, generally well tolerated, and compared favorably to historical trials with R-CHOP in DLBCL [87,88].

Liposomal cytarabine (DepoCyt^®^) was a liposomal formulation of cytosine arabinoside (ara-C), which was derived to overcome the main drawback of the free drug: its rapid degradation to non-active uracil derivatives during the circulation. In 1999, DepoCyt^®^ was approved for intrathecal treatment of lymphomatous neoplastic meningitis. In 2017, however, Pacira Pharmaceuticals announced the permanent discontinuation of DepoCyt injection due to persistent technical issues related to the product’s manufacturing process (https://professionals.optumrx.com/publications/library/drugwithdrawal-depocyt-2017-0706.html; accessed on 23 January 2022). Despite numerous attempts to develop a clinically approved cytarabine nanomedicine, the only ara-C nanodrug with marketing authorization is the above-mentioned liposomal combination of ara-C and daunorubicin (CPX-351) in patients with AML.

### 3.5. Targeted Liposomes

Similar to other nanoparticles, liposomes can be equipped with specific coupling ligands for targeted delivery to the tumor tissues. The active targeting of the liposomal nanomedicines should increase the efficacy of the tumor accumulation and consequently also the treatment efficacy of these formulations. Immunoliposomes that use monoclonal antibodies or their fragments for active targeting belong to the most promising nanodrugs [89]. Currently, transferrin receptor, epidermal growth factor receptor, and folate receptor belong to the most studied and employed cell targets for immunoliposomes. The targeting moiety (i.e., antibody, antibody fragment, or single-chain fragment variable (scFv)), the coupling method, and the rate of internalization of the liposome-receptor system all belong to critical factors that impact the anti-tumor efficacy of immunoliposomes. Recently, the clinical trials of immunoliposomes were reviewed in detail [90]. The results obtained so far demonstrate that immunoliposomes represent promising multivalent liposome-antibody constructs with unique therapeutic properties that can be used not only for the therapy but also for diagnostic or restaging procedures, e.g., in vivo imaging of circulating leukemia cells or visualization of tumor endothelium [90].

Beside the monoclonal antibodies, oligonucleotide-derived aptamers represent another class of promising targeting ligands. Aptamers, sometimes called “chemical antibodies” are small nucleic acid ligands composed of RNA or single-stranded DNA oligonucleotides that possess high specificity and affinity for their targets [91]. Their interaction with various targets is similar to that of antibodies: by recognizing a specific three-dimensional structure.

## 4. Polymer-Based Nanomedicines

### 4.1. PEG-Based Polymers

The most studied non-degradable water-soluble polymer used for the synthesis of polymer–drug conjugates is poly(ethylene glycol) (PEG) [92]. Monofunctional methoxy PEG is widely used for surface modification of proteins or liposomes to stealth them against biodegradation and improve their pharmacokinetics. Some of these conjugates successfully passed clinical evaluations and were granted marketing authorization, including pegaspargase (Oncaspar^®^) or peginterferon alfa-2a (Pegasys^®^). Pegaspargase is a polymer–drug conjugate used in the treatment of acute lymphoblastic leukemia (ALL) [93]. PEG stabilizes the asparaginase enzyme in the conjugate, which prolongs its circulation and reduces the blood levels of the amino acid asparagine. As an alternative to PEG, various poly(2-oxazoline) polymers (POx) were proposed and synthesized [94]. The use of POx enabled the introduction of various functional groups along the polymer backbone. Although POx are very promising materials for biomedical applications, they remain a domain of preclinical research [95,96].

### 4.2. HPMA-Based Polymers

**Figure 4:**
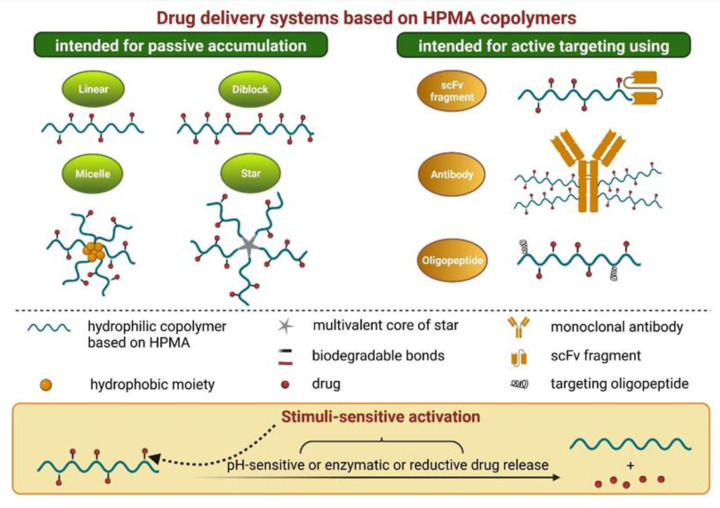
Overview of drug delivery systems based on HPMA co-polymers. Created with BioRender 10 December 2021.

Among others, water-soluble polymer carriers based on *N*-(2-hydroxypropyl) methacrylamide (HPMA) copolymers and their drug conjugates have been extensively studied because of their excellent in vitro and in vivo properties. The biocompatibility, non-toxicity, non-fouling properties, and apyrogenic character of HPMA copolymers were demonstrated and described in numerous studies [97]. Because of the limited clinical activity of first-generation HPMA-based nanomedicines, next-generation high-molecular-weight HPMA-based nanotherapeutics were designed with improved pharmacokinetic and biological properties. Various structures including diblocks, multiblocks, grafts, or star-like shapes were developed (Figure 4), and their enhanced treatment efficacy compared to the first-generation HPMA copolymers was confirmed in vivo on animal models of both solid tumors and hematological malignancies [98]. Besides the passive accumulation of the attached drugs achieved predominantly by the EPR effect, HPMA copolymers can be effectively modified as active targeting systems, most frequently by the conjugation of various monoclonal antibodies or their fragments [99] (Figure 4). In addition, HPMA-based nanomedicines can serve as effective theranostics [100].

## 5. Theranostics

### 5.1. The Origins and Evolution of the Theranostic Concept

Theranostics (or theragnostics) is a term with multiple definitions and interpretations. Its first use dates back to 1998 [101]. In nuclear medicine, it refers to a concept of combined targeted imaging and therapy delivery. The imaging allows both visualization of the tumor cells and estimation of the effectiveness of the administered therapy. The binding part of a radiopharmaceutical is targeted to a specific structure on tumor cells. The targeting vector is labeled with a beta-plus emitting radionuclide for PET (or a gamma emitter for a single-photon emission computed tomography, SPECT) for the purpose of characterization of the tumor tissue and for the confirmation of presence of the target molecule in the tumor mass. This can also provide more accurate information about disease spread than conventional imaging alone. If the expression of the target is sufficient, the same binding molecule can be labeled with a beta-minus or alpha emitter and used for therapy [102]. The imaging can eventually be used for dosimetric calculations.

Although the term “theranostics” has been used frequently in the last two decades, the practical use of this approach is much older. The first administration of the radioactive iodine ^131^I for the treatment of thyroid cancer was performed in 1946, and after the imaging technology was implemented into clinical practice, it became an integral part of thyroid cancer management [103,104]. Theranostics has emerged as a promising strategy for the treatment of selected solid tumors, and it is becoming a routine option in their management [105,106]. Theranostic strategies have a great potential in the management of patients with hematologic malignancies because of their predominant radiosensitivity and multifocal presentation [107].

### 5.2. Radioimmunotherapy (RIT)

Even though a plethora of theranostic pairs have been tested preclinically, the number of approved clinical applications remain limited. The concept of radioimmunotherapy (RIT) represents an example of a successful theranostics application in hematology. Compared to external-beam radiotherapy, RIT achieves a targeted delivery of a radionuclide bound to the monoclonal antibody directly to the lymphoma cells at distant (and distinct) anatomical sites. The so-called crossfire effect, mediated by the emitted electrons and gamma rays, leads also to eradication of lymphoma cells with no/low expression of the target antigen. There are two anti-CD20 radiopharmaceuticals approved for the treatment of B-cell lymphoma: A combined beta- and gamma-emitter iodine-131 tositumomab (Bexxar^®^) with a half-life of approx. 8 days and a pure beta-emitter yttrium-90 ibritumomab tiuxetan (Zevalin^®^) with a half-life of 2.7 days [108,109]. Both radiopharmaceuticals originally included a pre-therapeutic scintigraphic imaging step with lower activity for dosimetric purposes (labeling with ^131^I in the case of tositumomab, and ibritumomab was labeled with ^111^In). This step was later omitted from the ibritumomab therapy. However, due to complicated manufacture and administration procedures and with the advent of innovative targeted and immunotherapy agents, the clinical use of RIT in the management of B-NHL has become limited.

### 5.3. Pretargeted RIT

**Figure 5:**
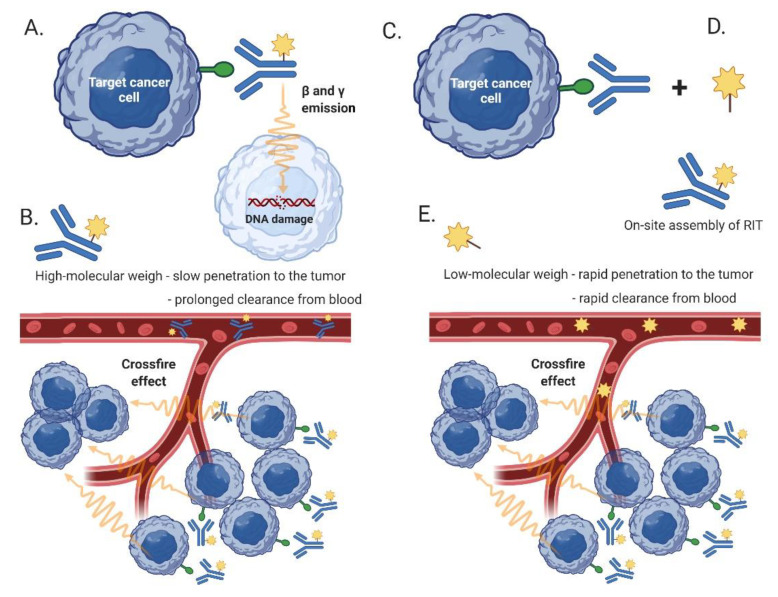
Mode of action of conventional and pretargeted radioimmunotherapy. (**A**) In conventional RIT, a radioimmunoconjugate binds directly to the tumor cells, but due to its high-molecular weight (**B**) it is slowly cleared from the bloodstream resulting in enhanced toxicity. (**C**) Compared to RIT, PRIT employs a targeting MAb as the first step, followed by the administration (**D**) of a small-molecular-weight radioconjugate that (**E**) rapidly penetrates to the tumor and that is rapidly cleared from the bloodstream, resulting in fewer off-target side effects. Created with BioRender 02 December 2021.

Compared to a single-step RIT, pretargeted RIT (PRIT) is a two-step process developed to address limitations of RIT, namely, to prevent long-term circulation of a radioactive RIT in the blood (Figure 5). In the first step of PRIT, a naked (targeting, cold) antibody is administered to the patient with the principal goal to bind all targeted antigens present on the tumor cells at all involved sites. In the second step (after the unbound targeting antibody has been cleared from the circulation) the patients are injected with a low-molecular-weight radioactive agent with high affinity to the targeting antibody. The small size of the radioactive agent is responsible both for rapid penetration of the agent to the involved organs and tissues (where it is bound to the targeting antibody) and for the rapid clearance of the free (unbound) agent from the circulation. The improved pharmacokinetics translate into improved efficacy and fewer side effects. Experimental PRIT targeting CD38 has demonstrated promising preclinical activity in vivo in experimental models of B-NHL [110,111].

### 5.4. Immuno-PET

The main limitation of the standard ^18^F-FDG-based PET is its incapability to detect metabolically inactive lymphoma cells. In contrast, MAb-based PET imaging (immuno-PET) approaches allow detection of all lymphoma cells irrespective of their metabolic activity [112]. An immuno-PET imaging probe consists of a targeting vector (usually a monoclonal antibody or its fragment) conjugated with a radiometal. Small-sized vectors (antibody fragments, nanobodies) are preferable due to their better tumor penetration and accelerated clearance from the bloodstream. The radiometals are loaded to the vector using either a random or site-specific conjugation [113]. Long half-life isotopes, such as cuprum-64 (^64^Cu, half-life 12 h) or zirconium-89 (^89^Zr, half-life 78 h), belong among the most commonly used radionuclides for this purpose [114]. In the context of NHL, immuno-PET was studied using antibodies targeting CD20 (e.g., rituximab, obinutuzumab), CD38 (daratumumab), CD30 (SGN-30), and others. It was repeatedly demonstrated that anti-CD20 antibodies or antibody fragments labeled with radiotracers enabled dynamic visualization of lymphoma cells and measurement of the delivered therapeutic effect in patients with NHL [115].

### 5.5. CXCR4-Targeted Therapy and Other Theranostic Concepts

Other published clinically tested approaches in hemato-oncology are rather scarce. The C-X-C chemokine receptor 4 (CXCR4, CD184) appears to be one of the most promising targets in hematologic malignancies and some solid tumors. The CXCR4-targeted peptide receptor radionuclide therapy employs the small cyclic pentapeptide ^68^Ga-Pentixafor for the sensitive and high-contrast imaging, while ^90^Y- or Lutetium-177 (^177^Lu)-pentixather (3-iodo-D-Tyr1-Pentixafor) with longer circulation times and delayed whole-body clearance is used for CXCR4-targeted therapy [116]. Clinical activity was demonstrated in small groups of patients with acute leukemias, advanced DLBCL, and MM [117].

Paul et al. performed an interesting pilot study that analyzed the treatment of four patients with malignancy (one with DLBCL) with ^18^F-fluorodeoxyglucose alone [118]. This concept breaks the traditional view of FDG as an option used for imaging only. Positrons emitted by ^18^F have similar features to electrons and can deliver cytotoxic energy to lymphoma cells. Although the primary objective of this study was safety, anti-tumor activity was documented.

Several other radiopharmaceuticals with a theranostic potential have been tested in numerous preclinical studies.

In conclusion, theranostics is an attractive approach to personalized medicine that is relatively well established in the management of some solid tumors. Except for RIT, however, other theranostic concepts in the field of hemato-oncology are confined mostly to the experimental (preclinical) phase.

## 6. Theranostic Strategies Based on Magnetic Resonance Imaging

Unlike in nuclear medicine, the tracer (contrast material) in magnetic resonance (MR) imaging (MRI) does not itself confer any anti-cancer effect whatsoever. Instead, it is used to visualize the distribution of the injected carrier substance, which may be loaded with an anti-cancer drug and enter the tumoral mass through passive or active targeting referred to as “tumor-targeted, MRI traceable nanotheranostics” [119]. Smart MR contrast agents result in selective enhancement in the tumor microenvironment not only by increased permeability but also by acid pH [120]. Contrast materials in MRI act by shortening longitudinal relaxation time (T1) or shortening transverse relaxation time (T2). The latter group is represented by superparamagnetic iron oxide nanoparticles. T1 contrast agents are typically gadolinium (Gd) chelates, some ultra-small superparamagnetic iron oxide nanoparticles (SPIONs), and manganese oxide nanoparticles.

### 6.1. Gadolinium-Based Tracers and Manganese Oxide Nanoparticles

Gadolinium-based contrast materials are used to visualize tissue enhancement by shortening T1 relaxation time and increasing signal intensity (bright signal). While Gd-based contrast materials are widespread, the preclinical evidence on theranostics using Gd imaging is very limited.

Shortening of T1 relaxation time can be achieved not only by Gd but also by manganese (Mn) oxide nanoparticles (MON) [121]. Recently, MONs became widely studied due to their negligible toxicity and bright signal on T1. Another advantage of MONs is that they can also interact with the tumor microenvironment in a non-specific manner (pH, reactive oxygen species, phagocytosis) [122]. MONs with triphenylphosphonium (PPh_3_) were experimentally used in HeLa cells to target mitochondrial function and induce cell death [6]. Manganese oxide together with chemotherapeutic drugs can also be loaded into micellar or polymerous nanospheres [123,124]. Preparation and experimental use of other Mn oxides in both MR imaging and targeted therapy have been also studied.

### 6.2. Magnetic Nanoparticles

Magnetic nanoparticles are based on ferromagnetic or ferrimagnetic metals, most commonly iron but also cobalt, nickel, and their compounds and oxides. These nanoparticles exhibit superparamagnetism. In the absence of an external magnetic field, their magnetization randomly flips its direction, and they rest in a paramagnetic state but have higher magnetic susceptibility. With the application of external magnetization, a single high magnetic moment is recruited from the moments of the whole ensemble of nanoparticles.

Superparamagnetic iron oxide nanoparticles (SPIONs) are widely studied due to their minimal toxicity. SPIONs have important properties suitable for targeted therapy. They can be heated by an alternating magnetic field or laser light, regionally distributed using an external magnetic field, and visualized by MRI. SPIONs also exhibit a degree of passive targeting through the EPR effect and can be coupled with epitopes for active targeting. Importantly, SPION nanoparticles can be loaded with chemotherapeutic or immunotherapeutic drugs. Although SPION compounds have demonstrated activity in the targeted anti-cancer treatment in cell cultures and animal models, their clinical use remains limited [125,126].

## 7. Conclusions

In the last two decades, the concept of anti-lymphoma therapy has dramatically changed with a gradual decline of standard cytostatic agents and rapid implementation of innovative targeted, biological, and immunotherapy agents with better anti-tumor efficacy and fewer side effects. Despite that, genotoxic agents still represent an indispensable backbone of anti-lymphoma therapy, especially of aggressive lymphoma subtypes. It appears that new formulation of “old” conventional cytostatics, with their passive and/or active delivery and controlled release, might represent the future of these highly effective anti-tumor drugs by improving their pharmacokinetics, increasing the therapeutic index, enhancing on-target efficacy, and reducing toxic side effects. Moreover, the binding of imaging probes to these nanomedical biomolecules might in the near future serve not only for more efficacious therapy but also for dynamic restaging procedures or the detection and eradication of the minimal residual disease.

## Figures and Tables

**Table 1:** Antibody–drug conjugates and immunotoxins approved for the therapy of lymphoproliferative malignancies.

Generic Name	Trade Name	Target Antigen	Linker	Toxic Payload	Target Patient Population	Approval Date
Brentuximab vedotin	Adcetris^®^	CD30	Enzyme cleavable	Auristatin	R/R HL, CD30+ T-NHL, MF	2017
Inotuzumab ozogamicin	Besponsa^®^	CD22	pH cleavable	Calicheamicin	R/R B-ALL	2017
Moxetumomab pasudotox	Lumoxiti^®^	CD22	Enzyme cleavable	Pseudomonas exotoxin	R/R HCL	2018
Polatuzumab vedotin	Polivy^®^	CD79B	Enzyme cleavable	Auristatin	R/R DLBCL	2019
Loncastuximab tesirine	Zynlonta^®^	CD19	Enzyme cleavable	PBD dimer	R/R DLBCL	2021

Abbreviations: B-ALL = B-cell acute lymphoblastic leukemia; DLBCL = diffuse large B-cell lymphoma; HCL = hairy cell leukemia; HL = Hodgkin lymphoma; R/R = relapsed/refractory.

## Abbreviations

ADC	ntibody drug conjugate
ADCC	antibody-dependent cell-mediated cytotoxicity
ADPC	antibody-dependent cell-mediated phagocytosis
ALL	acute lymphoblastic leukemia
AML	acute myeloid leukemia
BCMA	B-cell maturation antigen
BM	belantamab mafodotin
B-NHL	B-cell NHL
BV	brentuximab vedotin
CAR	chimeric antigen receptor
CAR19	CAR against CD19-positive cells
CDC	complement-dependent cytotoxicity
DAR	drug to antibody ration
DDS	drug delivery systems
DLBCL	diffuse large B-cell lymphoma
EPR	enhanced permeability and retention
FDG	2-[F^18^] fluoro-2-deoxy-D-glucose
FL	follicular lymphoma
Fv	fragment variable (of the monoclonal antibody)
Gd	gadolinium
GFLG	glycylphenylalanylleucylglycine
GO	gemtuzumab ozogamicin
HL	Hodgkin lymphoma
HPMA	*N*-(2-hydroxypropyl)methacrylamide
InO	inotuzumab ozogamicin
IT	immunotoxin
LT	loncastuximab tesirine
MCL	mantle cell lymphoma
MDR-1	multi-drug resistance protein 1
MM	multiple myeloma
MMAE	monomethyl auristatin E
MoA	mode of action
MRI	magnetic resonance imaging
NHL	non-Hodgkin lymphoma
NPLD	non-pegylated liposomal doxorubicin
MON	manganese oxide nanoparticles
MZL	marginal zone lymphoma
Mab	monoclonal antibody
NK	natural killer
PEG	polyethylene glycol
PGA	poly(L-glutamic acid)
PLD	pegylated liposomal doxorubicin
PET	positron emission tomography
PBDD	pyrrolobenzodiazepine dimer
Pox	poly(2-oxazoline) polymers
PRIT	pretargeted RIT
PSMA	prostate specific membrane antigen
PV	polatuzumab vedotin
R-CHOP	rituximab + cyclophosphamide + doxorubicin + vincristine + prednisone
RIT	radioimmunotherapy
R/R	relapsed/refractory
scFc	single-chain fragment variable
SPECT	single-photon emission computed tomography
SPION	superparamagnetic iron oxide nanoparticles
TDD	targeted drug delivery
T-NHL	T-cell NHL
VSLI	vincristine sulphate liposome injection
